# Quality of Life in a Saudi Arabian Sample of Parents and Caregivers of Children with Disabilities: A Cross-Sectional Analysis

**DOI:** 10.3390/healthcare14203525

**Published:** 2026-10-20

**Authors:** Abdullah Ahmed Alghamdi, Majed Ahmed Algarni, Mohammad S. Alzahrani

**Affiliations:** 1 Department of Special Education, College of Education, Taif University, Taif 21944, Saudi Arabia; 2 Department of Clinical Pharmacy, College of Pharmacy, Taif University, Taif 21944, Saudi Arabia; m.alqarni@tu.edu.sa (M.A.A.); m.s.alzahrani@tu.edu.sa (M.S.A.)

**Keywords:** quality of life, caregivers, children with disabilities, autism spectrum disorder, Saudi Arabia, cross-sectional studies

## Abstract

**Background**: Raising a child with a disability can affect multiple dimensions of caregiver quality of life (QoL), but caregiver experiences vary according to child, family, and service characteristics. **Objective**: The objective of this study was to assess QoL in a Saudi Arabian sample of parents and caregivers of children with disabilities using the Arabic Multicultural Quality of Life Index (MQLI-Ar) and to examine factors associated with total MQLI score. **Methods**: In this exploratory cross-sectional study, 336 adult parents/caregivers were recruited through specialized disability centers and associated parent networks using non-probability convenience sampling. QoL was measured with the 10-item MQLI-Ar. Bivariate associations were examined using *t*-tests/ANOVA, and adjusted associations were evaluated using multivariable linear regression. For the multivariable model, disability status was represented by mutually exclusive diagnostic profiles. **Results**: Among 336 participants, 75.9% were mothers and 60.1% resided in the Makkah region. The mean total MQLI score was 7.08 (SD = 2.28). The primary model was significant (F(15,320) = 2.89, *p* < 0.001, R^2^ = 0.119) (adjusted R^2^ = 0.078), and diagnostic profile was associated with MQLI score overall (*p* = 0.004). Relative to the autism-only group, higher adjusted scores were observed for learning difficulties only (B = 1.22, *p* = 0.009) and other disability only (B = 0.78, *p* = 0.030); the remaining diagnostic-profile contrasts were not statistically significant. **Conclusions**: Overall QoL was moderate to high in this caregiver sample, but QoL differed across diagnostic profiles. These exploratory cross-sectional findings should be interpreted cautiously given the non-probability sample, geographic concentration, modest model explanatory power, and unmeasured clinical and caregiving factors.

## 1. Introduction

Mental and neurodevelopmental conditions contribute substantially to health loss across the lifespan. The Global Burden of Disease Study 2023 estimated approximately 1.17 billion prevalent cases of mental disorders worldwide in 2023 across all ages and sexes, underscoring the broad population burden of conditions that often require sustained family and health-system support [1]. Raising a child with a disability can affect the physical, psychological, social, and financial well-being of parents and other family caregivers. Beyond the child’s health and developmental needs, caregivers may assume prolonged responsibilities related to treatment, rehabilitation, education, transportation, behavioral management, and coordination of healthcare and social services. These cumulative demands can affect multiple dimensions of caregivers’ quality of life (QoL), making caregiver well-being an important component of family-centered disability care [2,3,4]. Evidence across disability populations indicates that parents of children with neurodevelopmental and intellectual disabilities may experience lower QoL than parents of typically developing children, although the magnitude and domains affected vary according to child, caregiver, family, and environmental characteristics [4,5].

The association between childhood disability and caregiver QoL cannot be fully described by diagnosis alone. Child-related factors such as disability type, symptom severity, behavioral difficulties, functional dependency, and co-occurring disabilities may increase caregiving demands [3,6]. Parents of children with autism spectrum disorder (ASD; hereafter, autism), for example, frequently report challenges across physical, psychological, and social QoL domains, with greater disability severity and caregiving burden associated with poorer outcomes [7]. Similar concerns have been reported among families of children with attention-deficit/hyperactivity disorder (ADHD) and intellectual disability, for whom supervision, behavioral challenges, and dependency may place substantial demands on family functioning and caregiver well-being [4,8]. Contemporary neurodiversity frameworks also caution against interpreting neurodevelopmental differences exclusively through a deficit-based lens; caregiver well-being may instead reflect the interaction of individual support needs, functional challenges, environmental barriers, and access to appropriate services [9].

The broader social and healthcare context also shapes caregiver QoL. Financial resources, education, employment, social support, accessibility of healthcare, and availability of specialized services have all been associated with caregiver well-being [3,10]. Geographic distance and direct costs may impose additional burdens when care requires repeated visits or long-term multidisciplinary follow-up, whereas adequate social and disability-specific support may mitigate caregiving demands [6,10]. Family and parent factors also warrant explicit consideration across cultural settings because caregiving roles, social expectations, family structure, and access to formal and informal support can influence both child and caregiver outcomes [11].

These issues are particularly relevant for caregivers recruited in Saudi Arabia, where increasing attention has been directed toward the health and social needs of people with disabilities and their families. Recent Saudi studies have reported impaired QoL among caregivers of children with autism, ADHD, intellectual disability, and other disabilities [12,13,14]. A recent systematic review of Saudi evidence identified unfavorable outcomes across multiple QoL domains and highlighted socioeconomic disadvantage and restricted access to support services among factors associated with poorer caregiver well-being [10]. However, the literature remains heterogeneous: many studies focus on a single disability, are restricted to specific geographic regions, or use different QoL instruments, limiting comparisons across disability profiles and service contexts [10,12,13,14]. Relatively little Saudi evidence has simultaneously examined caregiver characteristics, multiple co-occurring disability types, medication use, geographic access to specialized care, payment status of services, and health insurance coverage within one analytical framework.

Reliable measurement is essential when examining these relationships. The Multicultural Quality of Life Index (MQLI) is a brief, self-administered instrument that provides a multidimensional and culturally informed assessment of QoL across physical and psychological well-being, independent and occupational functioning, interpersonal relationships, social and community support, personal and spiritual fulfillment, and overall perceived QoL [15]. The instrument has demonstrated favorable reliability and validity across cultural settings [15]. The Arabic version (MQLI-Ar) was subsequently translated, culturally adapted, and formally validated among parents and caregivers of children with disabilities in Saudi Arabia, demonstrating high internal consistency and satisfactory construct validity [16].

Accordingly, the present exploratory cross-sectional study aimed to assess QoL in a Saudi Arabian sample of parents and caregivers of children with disabilities using the MQLI-Ar and to examine caregiver-, child-, disability-profile-, and service-related factors associated with total MQLI score. Specifically, we evaluated caregiver sociodemographic characteristics, diagnostic profiles, current medication use, geographic access to specialized services, service payment status, and health insurance coverage. The objective was to characterize associations within the surveyed caregiver sample rather than to estimate nationally representative effects or causal relationships.

## 2. Methods

### 2.1. Study Design, Participants, and Setting

This exploratory cross-sectional study recruited adults aged 18 years or older who were parents or caregivers of a child with a diagnosed disability and resided in Saudi Arabia. Individuals who did not meet these eligibility criteria were excluded. Data were collected from 18 February 2024 to 22 June 2025 using non-probability convenience sampling. Recruitment was based on specialized health centers serving children with disabilities; the electronic survey link was distributed through healthcare workers at these centers and associated parent WhatsApp groups. The participating centers were included through convenience access rather than probability-based center sampling. Because dissemination occurred through multiple healthcare workers and parent networks, the total number of individuals who received or viewed the invitation could not be determined and a conventional response rate could not be calculated. All applicable survey questions required a response before submission; 336 completed questionnaires were received, none were excluded, and all 336 were included in the analysis. The survey did not impose a technical restriction limiting submissions to one response per device or account, and more than one caregiver of the same child could theoretically have participated. The survey was anonymous and collected no personally identifying information. Participation was voluntary, and electronic informed consent was obtained before survey completion. The study was approved by the Scientific Research Ethics Committee at Taif University (Approval No. 45-128; 16 January 2024). The present study began recruitment on 18 February 2024. The MQLI-Ar validation study [16] (N = 132) was initiated in January 2025 and used a separate survey link, so the two studies yielded separate response sets that were analyzed as separate datasets. However, both studies recruited through the same specialized disability centers, and their recruitment periods overlapped. The same caregivers may therefore have received both invitations and completed both surveys. Because both surveys were anonymous and collected no linking identifiers, the extent of any participant-level overlap cannot be determined. The two samples should therefore not be assumed to be independent.

### 2.2. Survey Tool

The questionnaire captured caregiver characteristics (relationship to the child, age group, education level, and family income), child characteristics (disability type, age at diagnosis, current age, birth order, and whether a sibling had also been diagnosed with a disability), and service-related factors (location of initial diagnosis, region of residence, distance to the nearest specialized care center, service payment status, health insurance coverage, and medication use). Child disability diagnoses were reported by caregivers and were not independently verified from medical records by the research team, although recruitment was conducted through specialized centers serving children with disabilities. Fee-based care was defined as services paid directly by the parent or family, whereas free care referred to government-funded services. Medication use referred to current disability-related medication use at the time of survey completion. Health insurance coverage referred specifically to coverage of the child’s healthcare, including medications.

Quality of life was assessed using the Multicultural Quality of Life Index (MQLI), a 10-item self-report instrument [15]. Each item rates a distinct QoL domain—physical well-being, psychological/emotional well-being, self-care and independent functioning, occupational functioning, interpersonal functioning, social–emotional support, community/services support, personal fulfillment, spiritual fulfillment, and overall perceived QoL—on a 0–10 numeric scale. The total MQLI score was calculated as the mean of all 10 items and was selected as the primary outcome because the instrument is designed to provide an overall multidimensional assessment while retaining domain-level descriptive information. The MQLI-Ar used in this study had previously undergone formal translation, cultural adaptation, and psychometric validation among parents and caregivers of children with disabilities in Saudi Arabia [16]. Because all survey items were mandatory, every participant completed all 10 MQLI items and no missing-item rule or prorated score was required.

### 2.3. Statistical Analysis

Sample characteristics are reported as frequencies and percentages. Caregiver and child characteristics were recoded where needed to avoid sparse categories: relationship to the child (Father/Mother/Sibling-Grandparent-Other), caregiver age (20–30/30–40/40–50/≥50 years), region (Riyadh/Makkah/Madinah/Other regions), and age at diagnosis (birth–6 years/≥7 years). Descriptive statistics (mean, standard deviation, and skewness) were calculated for each MQLI item and the total score. Current-sample reliability was assessed using Cronbach’s alpha, McDonald’s omega, corrected item-total correlations, and inter-item correlations; these analyses characterized reliability in the present sample rather than repeating the published validation of the MQLI-Ar [16].

Child disability type was collected as a multiple-response item. For descriptive and bivariate analyses, the original non-mutually exclusive indicators were retained (autism, ADHD, learning difficulties, intellectual disability, and other visual/physical/hearing/unspecified disabilities). For multivariable regression, disability status was re-parameterized into six mutually exclusive observed profiles: autism only (n = 95), ADHD only (n = 18), intellectual disability only (n = 50), learning difficulties only (n = 33), other disability only (n = 66), and multiple diagnoses (n = 74). Autism only, the largest single-diagnosis group, was used as the reference category. This parameterization ensured that all diagnostic contrasts were made between observed groups and avoided an unobserved all-zero diagnosis reference pattern.

Bivariate associations between total MQLI score and caregiver, child, and service-related characteristics were examined using independent-samples t-tests for two-group variables and one-way ANOVA for variables with more than two groups. Hedges’ g and mean differences with 95% confidence intervals were used to describe two-group effects, while eta-squared was used for multi-category comparisons. These analyses were exploratory, and no formal correction for multiple comparisons was applied; therefore, *p*-values close to 0.05 were interpreted cautiously. Because none of the multi-category variables showed a statistically significant omnibus ANOVA, post-hoc tests were not performed.

For the primary multivariable linear regression, non-diagnostic variables with bivariate *p*-values < 0.25 were considered using a purposeful-selection approach, while diagnostic profile was retained a priori as a single categorical factor. Because every child in the sample had at least one reported disability diagnosis and 78.0% had exactly one diagnosis, an all-zero diagnostic reference group did not exist. Therefore, disability status was represented by six mutually exclusive diagnostic profiles, with the autism-only group (n = 95) serving as the reference category. This approach allowed adjusted comparisons between observed diagnostic groups rather than relying on an unobserved reference pattern. Unstandardized regression coefficients (B) represented adjusted differences in total MQLI score on the 0–10 scale relative to the specified reference category.

Multicollinearity was assessed using variance inflation factors (VIFs). Model diagnostics included residual-versus-fitted plots, Q–Q plots, the Breusch–Pagan test, the Durbin–Watson statistic, and Cook’s distance. HC3 robust standard errors were examined as a sensitivity analysis. All statistical tests were two-tailed, with *p* < 0.05 considered statistically significant. Analyses were conducted using IBM SPSS Statistics, version 22, with supplementary diagnostics and HC3 estimates computed in SAS, version 9.4.

## 3. Results

### 3.1. Sample Characteristics

A total of 336 parents/caregivers were included in the analysis; all submitted questionnaires were complete and no responses were excluded. The sample was predominantly composed of mothers (75.9%) and participants residing in the Makkah region (60.1%) (Table 1). Nearly half of caregivers (49.7%) had a secondary education or less, family income was below 12,000 SAR/month for 81.0%, and 20.2% reported health insurance covering the child’s healthcare. Autism was the most frequently reported disability type (39.6%), followed by intellectual disability (21.7%), learning difficulties (20.8%), and ADHD (20.5%); 22.0% of children had two or more disability types recorded. For the mutually exclusive diagnostic profiles used in multivariable regression, 95 children had autism only, 18 ADHD only, 50 intellectual disability only, 33 learning difficulties only, 66 other disability only, and 74 multiple diagnoses. The uneven geographic distribution reflects the convenience recruitment strategy and should not be interpreted as nationally representative.

### 3.2. Quality-of-Life Domain Scores and Reliability

**Figure 1:**
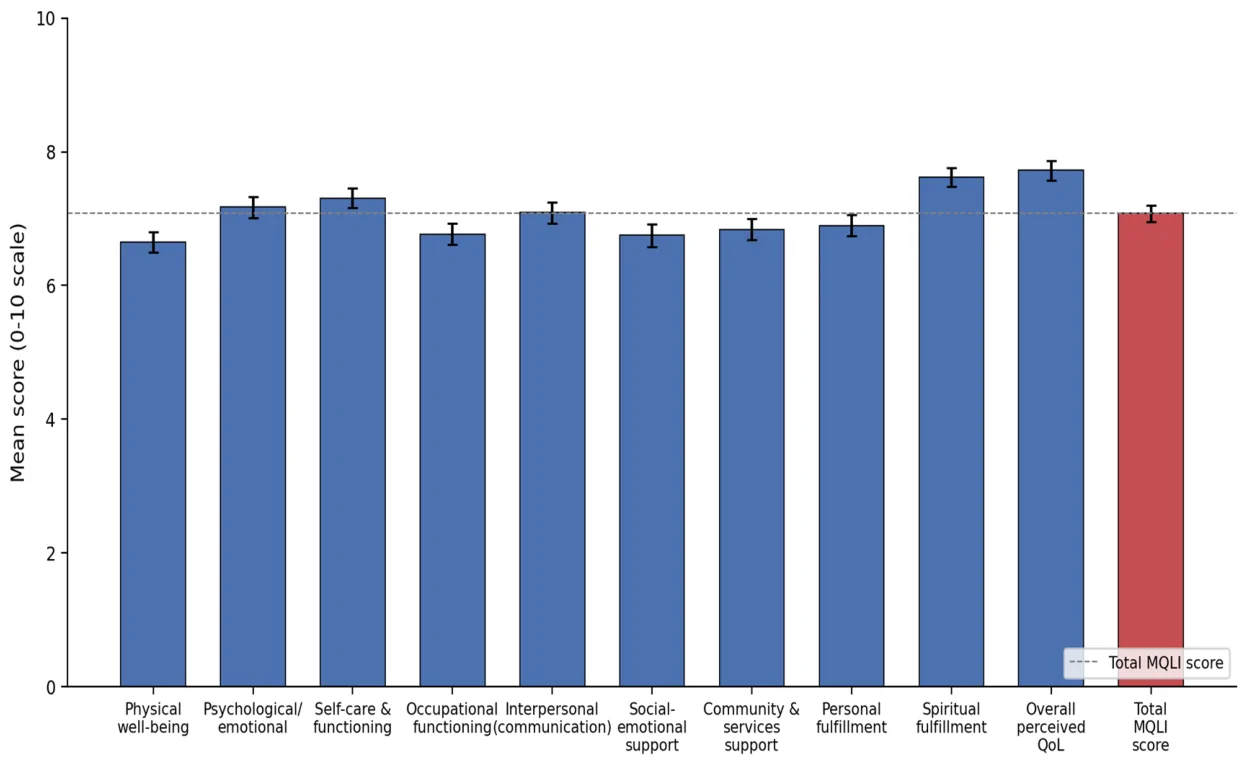
Mean scores across the ten MQLI domains, with standard error bars. Dashed line indicates the total MQLI score across all ten items.

The 10-item MQLI-Ar demonstrated high internal consistency in the present sample (Cronbach’s α = 0.941; McDonald’s ω = 0.941). Corrected item-total correlations ranged from 0.639 to 0.851, inter-item correlations ranged from 0.451 to 0.758 (mean = 0.613), and Cronbach’s alpha did not increase after deletion of any individual item (range 0.930–0.940). As shown in Table 2 and Figure 1, mean domain scores ranged from 6.65 for physical well-being to 7.72 for overall perceived QoL. The total MQLI score was 7.08 (SD = 2.28). Figure 1 compares the 10 domain means with the overall MQLI mean; physical well-being, social–emotional support, and occupational functioning were among the comparatively lower-scoring domains, whereas overall perceived QoL and spiritual fulfillment were the highest.

### 3.3. Quality of Life by Caregiver and Child Characteristics

Total MQLI scores differed significantly across several binary characteristics (Table 3). Caregivers of children using disability-related medication reported lower scores than non-users (M = 6.55 vs. 7.35, *p* = 0.002; Hedges’ g = −0.35), as did caregivers of children with ADHD (M = 6.39 vs. 7.26, *p* = 0.005; g = −0.38) or autism (M = 6.70 vs. 7.33, *p* = 0.014; g = −0.28). Scores were also lower among caregivers using fee-based rather than free care (M = 6.63 vs. 7.24, *p* = 0.030; g = −0.27) and among caregivers of children with two or more disability types (M = 6.60 vs. 7.22, *p* = 0.038; g = −0.27). The corresponding mean-difference 95% confidence intervals were approximately −1.31 to −0.29 for medication use, −1.47 to −0.27 for ADHD, −1.12 to −0.13 for autism, −1.17 to −0.06 for fee-based care, and −1.21 to −0.03 for two or more disability types. Effect sizes for multi-category comparisons were small (eta-squared approximately 0.003–0.015), and none of the multi-category omnibus ANOVAs was statistically significant; therefore, post-hoc testing was not performed. Because multiple exploratory comparisons were conducted without multiplicity adjustment, borderline *p*-values should be interpreted cautiously. The diagnosis-specific comparisons presented in Table 3 were unadjusted and based on non-mutually exclusive diagnostic categories. For each diagnosis, the “No” group comprised children with other reported disabilities rather than children without disabilities. Accordingly, these comparisons should not be interpreted as independent effects of individual diagnoses. Adjusted comparisons were instead based on the mutually exclusive diagnostic profiles used in the multivariable regression model.

### 3.4. Factors Associated with Total MQLI Score

**Figure 2:**
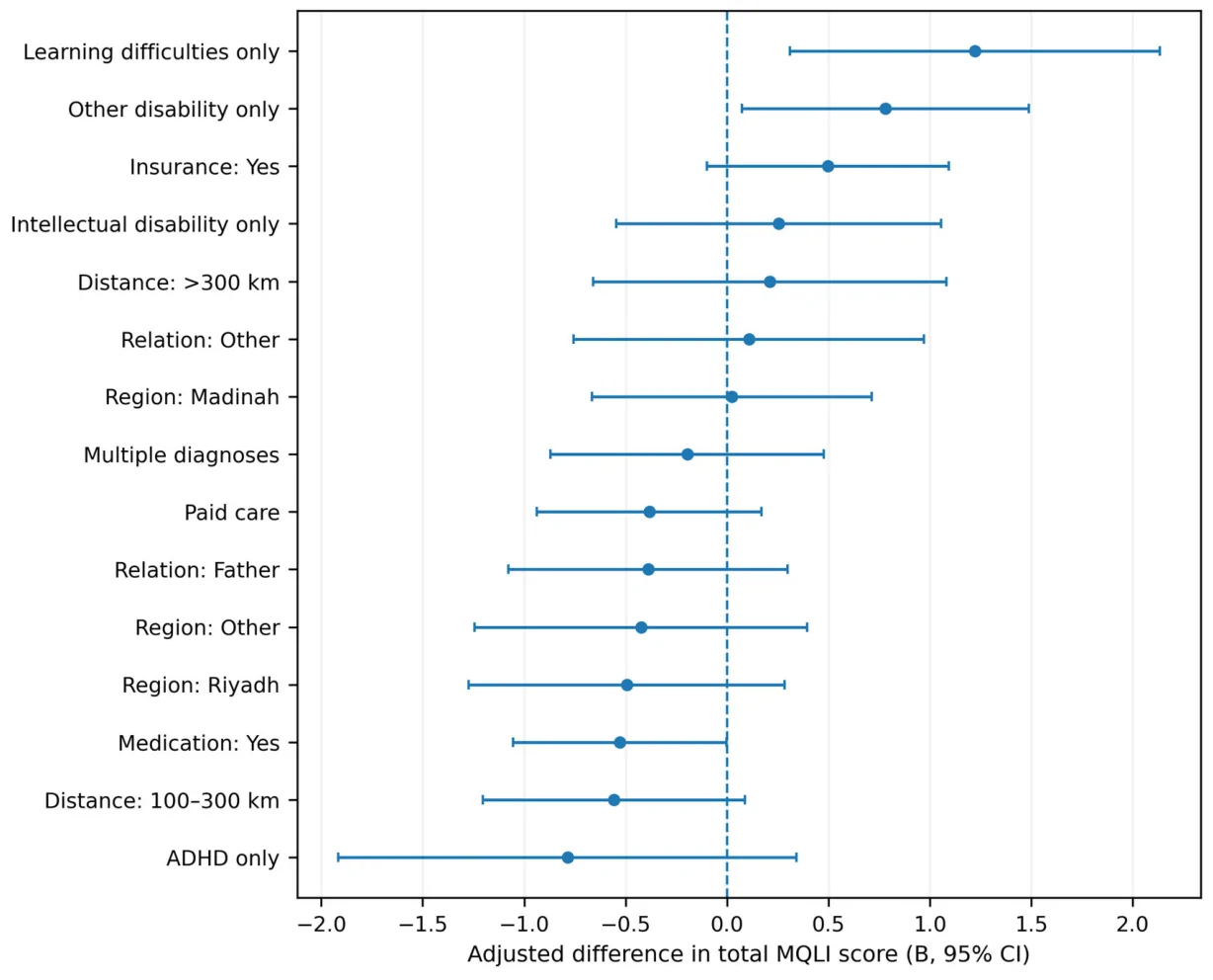
Adjusted associations with total MQLI score in the primary multivariable model. Points show unstandardized B coefficients and horizontal lines show 95% confidence intervals. For diagnostic-profile contrasts, autism only is the reference category; other reference categories are stated in Table 4. Confidence intervals crossing zero indicate *p* ≥ 0.05.

Table 4 presents unadjusted and adjusted linear regression analyses of total MQLI score, with adjusted coefficients from the primary model illustrated in Figure 2. All 336 participants were included and no observations were excluded because of missing model data. The primary model contained 15 predictor parameters and was statistically significant (F(15,320) = 2.89, *p* < 0.001, R^2^ = 0.119, adjusted R^2^ = 0.078). Diagnostic profile was associated with total MQLI score overall (partial F = 3.53, *p* = 0.004). Using autism only as the reference group, learning difficulties only (B = 1.22, 95% CI 0.31 to 2.13, *p* = 0.009) and other disability only (B = 0.78, 95% CI 0.07 to 1.49, *p* = 0.030) were associated with higher adjusted MQLI scores. ADHD only (B = −0.79, *p* = 0.172), intellectual disability only (B = 0.26, *p* = 0.530), and multiple diagnoses (B = −0.20, *p* = 0.568) did not differ significantly from autism only. Current medication use remained associated with a lower adjusted MQLI score (B = −0.53, 95% CI −1.05 to −0.001, *p* = 0.050).

The re-parameterized model showed little evidence of multicollinearity; VIFs for all predictors were below 1.5. Regression diagnostics showed no strong evidence of heteroscedasticity (Breusch–Pagan *p* = 0.068), acceptable residual independence (Durbin–Watson = 1.89), and no highly influential observations (maximum Cook’s distance = 0.046), although residuals showed some negative skewness. HC3 robust standard errors produced similar conclusions: the learning difficulties only and other disability only contrasts remained statistically significant, while the remaining diagnostic-profile contrasts remained non-significant.

## 4. Discussion

The present exploratory cross-sectional study examined QoL among parents and caregivers of children with disabilities in a Saudi Arabian caregiver sample using the culturally adapted MQLI-Ar. Overall, the mean MQLI score was 7.08 out of 10, but substantial variability was evident across participants. In the primary adjusted model, diagnostic profile was associated with total MQLI score overall. Because diagnostic status was modeled using mutually exclusive observed profiles, the coefficients should be interpreted as contrasts between diagnostic profiles rather than as independent effects of individual diagnoses.

Despite the demands associated with caring for a child with a disability, many participants reported comparatively positive overall QoL. This should not be interpreted as an absence of burden. Rather, multidimensional QoL can include relatively preserved domains alongside substantial practical or emotional challenges. Physical well-being, social–emotional support, occupational functioning, and community/services support were among the lower-scoring domains, whereas spiritual fulfillment and overall perceived QoL were the highest. This pattern reinforces the value of examining QoL as a multidimensional construct rather than relying on diagnostic status alone.

Spiritual fulfillment was among the highest-rated MQLI domains. Within the Saudi context, religious beliefs, spirituality, family cohesion, and extended-family networks may constitute important resources for some caregivers, although these mechanisms were not directly measured in the present study. Cross-cultural research similarly indicates that family and parent factors can shape mental-health and caregiving outcomes in ways that vary across settings [11]. Thus, similarities in caregiving demands across countries may coexist with cultural differences in family roles, informal support, service expectations, and the meaning assigned to disability and caregiving.

The re-parameterized analysis provides a more interpretable comparison across observed diagnostic profiles. Relative to caregivers of children in the autism-only group, caregivers in the learning-difficulties-only and other-disability-only groups had higher adjusted MQLI scores, whereas the ADHD-only, intellectual-disability-only, and multiple-diagnosis groups did not differ significantly from the autism-only group. These contrasts should not be interpreted as a ranking of diagnoses or as evidence that a diagnosis itself causes caregiver QoL differences. Diagnostic labels may proxy differences in functional dependence, behavioral difficulties, support needs, service access, and other clinical or family characteristics that were not measured.

The autism-only group was used as the reference because it was the largest single-diagnosis profile, allowing stable estimation of contrasts. Previous reviews and Saudi studies have documented challenges in physical, psychological, and social QoL among parents of children with autism [7,12,17,18]. A neurodiversity-informed interpretation remains important: differences in caregiver QoL should not be understood as evidence that neurodivergence is inherently burdensome, but rather as potentially reflecting interactions among support needs, functional and behavioral characteristics, environmental barriers, family resources, and service availability [9].

The ADHD-only group was relatively small (n = 18), and its adjusted estimate was imprecise. Although ADHD-related behavioral dysregulation, inattention, impulsivity, school difficulties, and comorbidity may increase supervision requirements and disrupt family routines [8,19], the present profile-based model did not demonstrate a statistically significant adjusted difference between ADHD only and autism only. Likewise, the intellectual-disability-only and multiple-diagnosis profiles did not differ significantly from the autism-only reference group. These findings reinforce the need to avoid attributing caregiver QoL directly to diagnostic category without measures of severity, functional dependence, behavioral burden, and caregiving intensity.

The mutually exclusive profile parameterization also resolved the interpretive and collinearity concerns created by simultaneously entering overlapping diagnosis indicators and a derived multiple-diagnosis indicator. In the revised model, all diagnostic coefficients compare observed groups, and VIFs were below 1.5. Nevertheless, broad profile categories cannot capture the clinical heterogeneity within each diagnosis or the specific combinations represented in the multiple-diagnosis group. Future research should incorporate direct measures of clinical complexity, severity, functional dependency, and specific co-occurring conditions.

Current disability-related medication use was associated with lower MQLI scores in the bivariate analysis and remained marginally associated after adjustment in the revised model. Medication use is unlikely to have a simple causal relationship with caregiver QoL and may instead function as a marker of greater symptom burden, clinical complexity, or treatment intensity. Information on medication indication, duration, effectiveness, adverse effects, and adherence was not collected; consequently, this association should be interpreted cautiously.

Several service-related characteristics were associated with caregiver QoL at the bivariate level but not after adjustment. Fee-based care was associated with a modestly lower unadjusted MQLI score, while distance to specialized care and insurance coverage showed non-significant differences. These patterns are clinically plausible because repeated visits, transportation, time away from employment, and direct payment for rehabilitation or disability-related services may create financial and logistical strain. Saudi evidence has previously identified socioeconomic disadvantage and restricted access to services as correlates of poorer caregiver QoL [10]. Because the present bivariate analyses were exploratory and multiple comparisons were not adjusted for multiplicity, these service-related findings should not be overinterpreted.

Health insurance coverage was reported by only about one-fifth of participants and was associated with a higher mean MQLI score, although the difference was not statistically significant in the primary analyses. Insurance status may not fully represent financial protection for families of children with disabilities, because rehabilitation, educational, behavioral, and social-support services may not be captured adequately by conventional coverage. Future studies should assess actual service utilization, benefit coverage, unmet needs, and out-of-pocket expenditure.

Caregiver age, education, and household income were not significantly associated with QoL in this sample. This differs somewhat from the recent Saudi systematic review, which identified lower educational attainment and socioeconomic disadvantage as recurring correlates of poorer caregiver QoL [10]. Differences may reflect sampling, measurement, broad category definitions, or indirect pathways through service access, employment disruption, social support, or disability severity. These findings emphasize the context-dependent and multifactorial nature of caregiver QoL.

The final primary model explained 11.9% of the variance in total MQLI score (adjusted R^2^ = 7.8%), indicating that most variability in caregiver QoL was not captured by the measured characteristics. Accordingly, the observed diagnostic-profile and medication associations should be viewed as modest components of a broader multifactorial outcome. Important unmeasured factors may include disability severity, functional dependence, behavioral problems, duration and intensity of caregiving, sleep disturbance, caregiver depression or anxiety, social support, family functioning, employment and income security, service quality, and out-of-pocket expenditure. Residual confounding by these factors may partly account for the observed associations.

The findings may nevertheless help identify caregiver groups who warrant further assessment. Family-centered disability services could incorporate routine evaluation of caregiver well-being and facilitate access to psychosocial, social, educational, and practical support. Evidence suggests that peer-support interventions can improve aspects of caregiver well-being, although effectiveness varies across populations and settings [20]. Clinical and service responses should therefore be guided by individual family needs and functional caregiving demands rather than diagnosis alone.

### Strengths and Limitations

This study has several strengths. It examined multiple disability categories within the same sample, used mutually exclusive observed diagnostic profiles for the multivariable analysis, used the previously validated MQLI-Ar [16], and considered caregiver, child, disability-profile, and service-related characteristics within one analytical framework. Reliability analyses, regression diagnostics, effect-size estimates, and robust-standard-error sensitivity analyses were undertaken to improve transparency and assess the robustness of the primary findings.

Several limitations should be acknowledged. First, the cross-sectional design precludes conclusions about temporality or causality. Second, non-probability convenience recruitment through specialized disability centers and associated parent networks may have introduced selection bias and may underrepresent families with limited access to clinical centers, internet services, or active parent networks. The invitation denominator was unknown, so a response rate could not be calculated. Third, 60.1% of participants resided in the Makkah region, and relatively small numbers from other regions limit national generalizability and reliable regional comparisons; the findings therefore pertain to the surveyed Saudi Arabian caregiver sample rather than the national population. Fourth, the survey did not technically restrict submissions to one device or account, and more than one caregiver of the same child could theoretically have participated, so complete independence at the family level could not be verified. Fifth, although recruitment was conducted through specialized centers serving children with disabilities, questionnaire data were caregiver-reported and may therefore be subject to reporting and recall bias; diagnoses were not independently verified from medical records by the research team. Sixth, the MQLI-Ar validation study used a separate survey link but recruited through the same centers during an overlapping period; because both surveys were anonymous, some caregivers may have completed both, and the extent of overlap cannot be quantified. Seventh, the primary model used exploratory screening for non-diagnostic covariates and explained only a modest proportion of QoL variance. Finally, key factors such as disability severity, functional dependence, behavioral symptoms, caregiving intensity, caregiver mental health, social support, and detailed financial burden were not measured. These limitations warrant cautious interpretation and should guide more representative, longitudinal, and clinically detailed future research.

## 5. Conclusions

In this Saudi Arabian caregiver sample, overall QoL was moderate to high, and total MQLI scores varied across observed diagnostic profiles. In the adjusted model, diagnostic profile was associated with MQLI score overall; compared with the autism-only reference group, higher adjusted scores were observed for the learning-difficulties-only and other-disability-only groups, while the remaining profile contrasts were not statistically significant. These exploratory cross-sectional associations should not be interpreted as causal effects or as a ranking of diagnoses. Interpretation is constrained by the non-probability sample, geographic concentration, modest explanatory power of the model, potential reporting bias, and unmeasured clinical and caregiving factors. Future studies should use more representative and longitudinal designs and incorporate direct measures of disability severity, functional dependence, behavioral difficulties, caregiving intensity, caregiver mental health, social support, service quality, and financial strain.

## Figures and Tables

**Table 1:** Sample characteristics (N = 336).

Characteristic	n	%
**Caregiver Characteristics**		
**Relationship to the disabled child**		
Father	51	15.2%
Mother	255	75.9%
Sibling/grandparent/other	30	8.9%
**Age**		
20–29 years	43	12.8%
30–39 years	126	37.5%
40–49 years	129	38.4%
50 years or older	38	11.3%
**Education level**		
High school or less	167	49.7%
Bachelor’s degree or equivalent	140	41.7%
Higher education	29	8.6%
**Family’s income**		
Less than 6000 SAR	134	39.9%
6000–12000 SAR	138	41.1%
More than 12000 SAR	64	19.0%
**Child Characteristics**		
**Child’s current age**		
1–6 years	77	22.9%
7–12 years	130	38.7%
13–18 years	76	22.6%
18 years and above	53	15.8%
**Child’s age at diagnosis**		
Birth to 6 years	293	87.2%
7 years or older	43	12.8%
**Birth order**		
First born	90	26.8%
Not the 1st born	246	73.2%
**Having a sibling diagnosed with the same or a different disability**		
No	277	82.4%
Yes	59	17.6%
**Child disability type: learning difficulties**		
No	266	79.2%
Yes	70	20.8%
**Child disability type: hyperactivity/attention deficit (ADHD)**		
No	267	79.5%
Yes	69	20.5%
**Child disability type: intellectual disability**		
No	263	78.3%
Yes	73	21.7%
**Child disability type: autism**		
No	203	60.4%
Yes	133	39.6%
**Child disability type: other (visual/physical/hearing/other)**		
No	251	74.7%
Yes	85	25.3%
**Child has 2+ disability types**		
No (1 diagnosis)	262	78.0%
Yes (2+ diagnoses)	74	22.0%
**Child use medication for the disability**		
No	224	66.7%
Yes	112	33.3%
**Service-Related Characteristics**		
**Region**		
Riyadh Region	39	11.6%
Makkah Region	202	60.1%
Madinah Region	59	17.6%
Other regions	36	10.7%
**Distance to the nearest center providing care for the child’s disability**		
100 km or less	250	74.4%
100–300 km	57	17.0%
More than 300 km	29	8.6%
**Is this center paid or free?**		
Free	249	74.1%
Paid	87	25.9%
**Do you have insurance that covers the child’s healthcare?**		
No	268	79.8%
Yes	68	20.2%

**Table 2:** Descriptive statistics for the ten MQLI domains.

Domain	Mean	SD	Skew
Physical well-being	6.65	2.77	−0.38
Psychological/emotional well-being	7.17	2.84	−0.73
Self-care and independent functioning	7.31	2.77	−0.80
Occupational functioning	6.77	2.86	−0.53
Interpersonal functioning (communication)	7.09	2.91	−0.69
Social–emotional support	6.75	3.11	−0.57
Community and services support	6.84	2.91	−0.57
Personal fulfillment (accomplishment)	6.90	2.81	−0.53
Spiritual fulfillment (religious life)	7.62	2.58	−1.03
Overall perceived quality of life	7.72	2.73	−1.05
**Total** **MQLI score (mean of 10 items)**	**7.08**	**2.28**	**−0.67**

Note. All items scored 0–10 (observed range 1–10 in this sample) [SD, standard deviation].

**Table 3:** Overall mean MQLI score by caregiver, child, and service characteristics.

Characteristic	Mean MQLI Score	SD	*p*
**Caregiver Characteristics**			
**Relationship to the disabled child**			0.204
Father	6.56	2.46	
Mother	7.18	2.24	
Sibling/grandparent/other	7.14	2.33	
**Age**			0.412
20–30 years	7.26	2.26	
30–40 years	6.81	2.47	
40–50 years	7.22	2.11	
50 years or older	7.31	2.20	
**Education level**			0.483
High school or less	7.03	2.42	
Bachelor’s degree or equivalent	7.22	2.18	
Higher education	6.69	1.95	
**Family’s income**			0.606
Less than 6000 SAR	7.19	2.35	
6000–12,000 SAR	6.93	2.27	
More than 12,000 SAR	7.17	2.17	
**Child Characteristics**			
**Child’s current age**			0.290
1–6 years	7.18	2.34	
7–12 years	6.99	2.15	
13–18 years	6.81	2.44	
18 years and above	7.56	2.28	
**Child’s age at diagnosis**			0.504
Birth to 6 years	7.05	2.30	
6 years or older	7.30	2.20	
**Birth order**			0.349
First born	6.89	2.40	
Not the 1st born	7.15	2.24	
**Having a sibling diagnosed with the same or a different disability**			0.868
No	7.09	2.26	
Yes	7.04	2.40	
**Child disability type: learning difficulties**			0.116
No	6.98	2.23	
Yes	7.46	2.45	
**Child disability type: hyperactivity/attention deficit (ADHD)**			**0.005**
No	7.26	2.24	
Yes	6.39	2.34	
**Child disability type: intellectual disability**			0.536
No	7.12	2.21	
Yes	6.94	2.55	
**Child disability type: autism**			**0.014**
No	7.33	2.30	
Yes	6.70	2.22	
**Child disability type: other (visual/physical/hearing/other)**			0.487
No	7.05	2.31	
Yes	7.30	2.14	
**Child has 2+ disability types**			**0.038**
No (1 diagnosis)	7.22	2.23	
Yes (2+ diagnoses)	6.60	2.40	
**Child use medication for the disability**			**0.002**
No	7.35	2.28	
Yes	6.55	2.20	
**Service-Related Characteristics**			
**Region**			0.233
Riyadh Region	6.75	2.49	
Makkah Region	7.11	2.32	
Madinah Region	7.48	1.93	
Other regions	6.61	2.30	
**Distance to the nearest center providing care for the child’s disability**			0.079
100 km or less	7.21	2.26	
100–300 km	6.46	2.51	
More than 300 km	7.19	1.90	
**Is this center paid or free?**			**0.030**
Free	7.24	2.28	
Paid	6.63	2.26	
**Do you have insurance that covers the child’s healthcare?**			0.075
No	6.97	2.31	
Yes	7.52	2.14	

**Table 4:** Unadjusted and adjusted linear regression of total MQLI score.

Variable	Unadjusted B (95% CI)	*p*	Adjusted B (95% CI)	*p*
**Relation to child (ref = mother)**				
Father	−0.62 (−1.31, 0.07)	0.076	−0.39 (−1.08, 0.30)	0.267
Sibling/grandparent/other	−0.04 (−0.90, 0.83)	0.934	0.11 (−0.76, 0.97)	0.806
**Region (ref = Makkah)**				
Riyadh	−0.37 (−1.15, 0.42)	0.356	−0.49 (−1.27, 0.29)	0.213
Madinah	0.37 (−0.29, 1.03)	0.273	0.02 (−0.66, 0.71)	0.944
Other regions	−0.51 (−1.32, 0.30)	0.218	−0.42 (−1.24, 0.40)	0.309
**Distance to nearest care center (ref = ≤100 km)**				
100–300 km	−0.75 (−1.41, −0.09)	**0.025**	−0.56 (−1.20, 0.09)	0.091
>300 km	−0.02 (−0.90, 0.86)	0.968	0.21 (−0.66, 1.08)	0.635
**Care center is fee-based (ref = free)**				
Paid	−0.62 (−1.17, −0.06)	**0.030**	−0.38 (−0.94, 0.17)	0.175
**Has health insurance for child’s care (ref = no)**				
Yes	0.55 (−0.06, 1.16)	0.075	0.50 (−0.10, 1.09)	0.102
**Child uses medication (ref = no)**				
Yes	−0.80 (−1.31, −0.29)	**0.002**	−0.53 (−1.05, −0.001)	0.050
**Diagnostic profile (ref = autism only)**				
ADHD only	−0.84 (−1.96, 0.28)	0.143	−0.79 (−1.92, 0.34)	0.172
Intellectual disability only	0.46 (−0.31, 1.22)	0.239	0.26 (−0.54, 1.06)	0.530
Learning difficulties only	1.49 (0.60, 2.37)	0.001	1.22 (0.31, 2.13)	0.009
Multiple diagnoses	−0.19 (−0.87, 0.49)	0.577	−0.20 (−0.87, 0.48)	0.568
Other disability only	0.84 (0.14, 1.54)	0.018	0.78 (0.07, 1.49)	0.030

## Data Availability

The data supporting the findings of this study are available from the corresponding author upon reasonable request. Access to the data may be granted for legitimate academic or research purposes, subject to ethical and privacy considerations.
